# Induction of Apoptotic Cell Death in Human Leukemia U937 Cells by C18 Hydroxy Unsaturated Fatty Acid Isolated from Red Alga *Tricleocarpa jejuensis*

**DOI:** 10.3390/md19030138

**Published:** 2021-03-02

**Authors:** Shijiao Zha, Mikinori Ueno, Yan Liang, Seiji Okada, Tatsuya Oda, Fumito Ishibashi

**Affiliations:** 1 Graduate School of Fisheries and Environmental Sciences, Nagasaki University, 1-14 Bunkyo-machi, Nagasaki 852-8521, Japan; bb53418001@ms.nagasaki-u.ac.jp (S.Z.); bb53419804@ms.nagasaki-u.ac.jp (Y.L.); t-oda@nagasaki-u.ac.jp (T.O.); 2 Joint Research Center for Human Retrovirus Infection, Division of Hematopoiesis, Kumamoto University, 2-2-1 Honjo, Chuo-ku, Kumamoto 860-0811, Japan; mikiueno@kumamoto-u.ac.jp (M.U.); okadas@kumamoto-u.ac.jp (S.O.)

**Keywords:** *Tricleocarpa jejuensis*, red alga, U937 cells, C18 hydroxy unsaturated fatty acid, apoptosis, caspases

## Abstract

Our previous studies have found that (±)-(*E*)-12-hydroxyoctadec-10-enoic acid (HOEA) isolated from the red alga *Tricleocarpa jejuensis* showed cytotoxic effects on various living organisms including harmful microalgae, Gram-positive bacteria, and mammalian tumor cells. Since natural products with apoptosis-inducing ability can be promising anti-cancer agents, in this study, we investigated the cytotoxic mechanism of HOEA on U937 cells focusing on apoptosis induction. HOEA showed much stronger cytotoxic and cytolytic effects on U937 cells than elaidic acid, which has similar structure but no 12-hydroxy group, suggesting that hydroxy group is important for the cytotoxicity of HOEA. HOEA induced apoptotic nuclear morphological changes, DNA fragmentation, and decrease in mitochondrial membrane potential. Furthermore, time-dependent increase in annexin V+/PI+ cell population in HOEA-treated U937 cells was detected. Among the apoptosis-related reagents, caspase-family inhibitor almost completely inhibited HOEA-induced DNA fragmentation. In the analyses using specific caspase-substrates, extremely high cleavage activity toward caspase-3/7/8 substrate was observed in HOEA-treated U937 cells, and weak activities of caspase-1 and -3 were detected. Analyses using specific caspase inhibitors suggested that caspase-3 and caspase-8 might be predominantly responsible for the cleavage activity. Activation of these caspases were also confirmed by western blotting in which significant levels of cleaved forms of caspase 3, caspase 8, and PARP were detected in HOEA-treated U937 cells. Our results suggest that HOEA is capable of inducing apoptosis in U937 cells in which caspase-3 and caspase-8 might play important roles. Since the cytotoxic effect of HOEA is not strictly specific to tumor cells, development of appropriate drug delivery system for selective tumor targeting is necessary for the clinical applications to reduce the possible side effects.

## 1. Introduction

Marine algae are attractive rich sources of natural compounds with various biological activities. Some of them show bactericidal [1], anti-cancer [2], antioxidant [3], anti-inflammatory [4], and anti-microalgal [5] activities depending on the molecular structures and their sources. Since harmful algal blooms (HAB) have been continuously causing huge detrimental impact on marine environment and marine organisms including aqua-cultured fish and shellfish, our research interests have been mainly concentrated on the seeking of effective algicidal agents which can be applied to the marine field and are urgently required. Considering the advantage of the environmentally friendly nature, macroalgae are ideal sources for the effective algicidal agents. Previous extensive studies have discovered diverse chemical compounds with growth inhibitory effects on HAB species from several marine macroalgal species. For instance, hydrophobic lipid-related [6,7,8,9,10,11,12], terpenoid [13,14,15,16,17], and phenolic compounds [13,17] have been found as algicidal agents. We previously screened algicidal compounds in more than 15 different species of macroalgae including red (Rhodophyta), brown (Phaeophyta), and green (Chlorophyta) algae harvested from the coastal areas of Kyushu in Japan. We eventually found that the red alga *Tricleocarpa jejuensis* had potent cytolytic compounds on *Chattonella antiqua* which is known as the most harmful red tide phytoplankton causing mass mortality of various fish species in Japan and other countries [18]. We conducted the structure evaluation of the active compounds by NMR, IR and mass spectral analyses, and found that the compounds were a mixture of four isomers of C18 hydroxy unsaturated fatty acids, which were only different in the position of the double bond each other. Their authentic molecules obtained by unambiguous syntheses confirmed the structures [18]. A typical one is (*E*)-12-hydroxyoctadec-10-enoic acid (HOEA). Several intermediate derivatives were obtained during the processes of the chemical syntheses. Comparative study on the synthetic intermediate derivatives and authentic compounds demonstrated that hydroxy group is important for the algicidal activity. Based on the results, it was concluded that C18 monounsaturated fatty acid with a hydroxy group is a promising algicidal agent. To further evaluate the bioactivities, we examined the effects of HOEA on various species including various harmful marine microalgae, Gram-positive and negative bacteria, and mammalian tumor cell lines. Interestingly, we found that HOEA was highly cytotoxic against tumor cells. Thus, HOEA is a promising multifunctional candidate with potential to be developed not only as anti-microalgae but also as anti-cancer chemotherapeutic drug [19]. 

Regarding anti-tumor compounds derived from marine algae, a wide-variety of compounds have been discovered from different algal sources, and some of them exhibit pro-apoptotic activity [20]. In cancer chemotherapy, acquisition of the ability of cancer cells to escape from apoptosis is one of the serious problem to overcome. Such ability contributes not only to tumor growth, but also to the metastasis and resistance to anti-cancer drugs. Therefore, bioactive compounds with the ability to induce apoptotic pathways directly in cancer cells can be a promising candidate for the development of cancer chemotherapeutic drugs. To ascertain whether or not the HOEA can induce apoptosis in tumor cells, in this study, we investigated the apoptotic features of human leukemia U937 cells after treatment with HOEA, and compared the activities with elaidic acid, a similar C18 monounsaturated fatty acid but lacking hydroxy group.

## 2. Results

### 2.1. Chemical Structure of HOEA and Elaidic Acid

**Figure 1:**
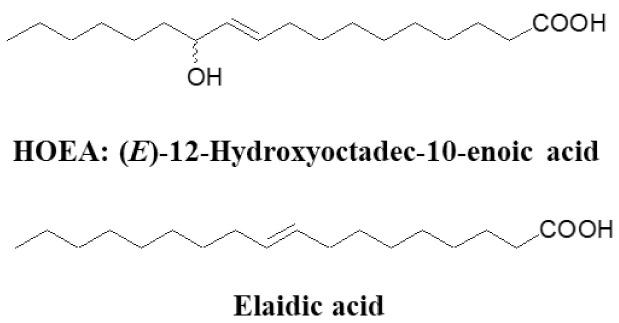
Structure of (*E*)-12-hydroxyoctadc-10-enoic acid (HOEA) and elaidic acid.

Figure 1 shows the structure of HOEA and elaidic acid. Elaidic acid is a commercially available monounsaturated C18 fatty acid and is structurally similar to HOEA except for the lack of hydroxy group and different position of double bond. To evaluate the importance of hydroxy group of HOEA for the cytotoxic potential on tumor cells, comparative studies between HOEA and elaidic acid were carried out.

### 2.2. Cytotoxicity of HOEA and Elaidic Acid on U937 Cells

**Figure 2:**
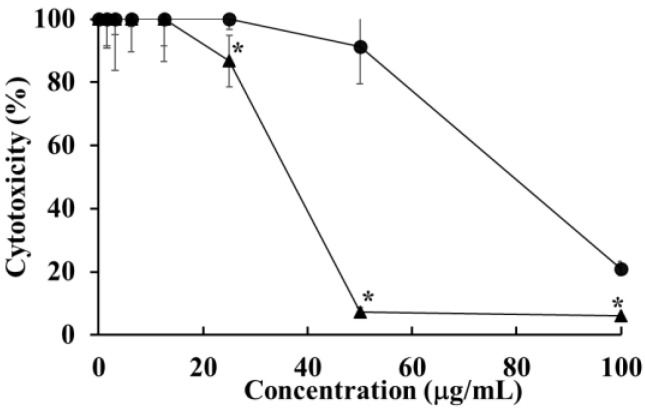
Cytotoxicity of HOEA (▲) and elaidic acid (●) on U937 cells. Cells in 96-well plates (2 × 10^4^ cells/well) were incubated with varying concentrations of HOEA or elaidic acid in the growth medium at 37 °C for 24 h, and then the viabilities of the cells were assessed by CCK-8 assay as described in the text. The points indicate the means of triplicate measurements and the bars indicate standard deviation. * indicate significant difference between HOEA and elaidic acid (*p* < 0.05).

The effects of HOEA and elaidic acid on the viability of U937 cells were examined by CCK-8 assay after 24 h incubation. The HOEA and elaidic acid showed cytotoxic effect on the cells in a concentration-dependent manner, and the IC_50_ was estimated to be 47.96 and 84.48 μg/mL, respectively (Figure 2), indicating that the cytotoxic potency of HOEA is much stronger than elaidic acid.

### 2.3. Cytolytic Effects of HOEA and Elaidic Acid on U937 Cells

**Figure 3:**
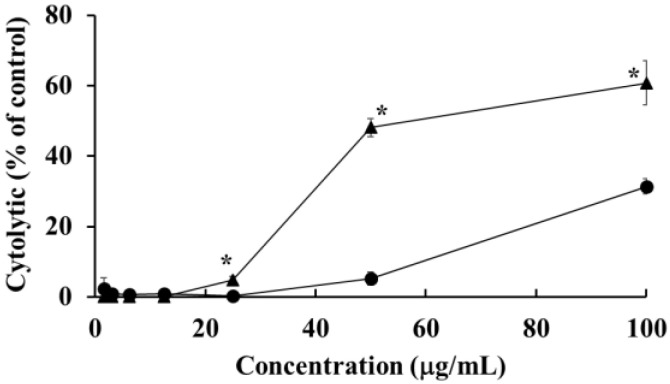
Cytolytic effects of HOEA (▲) and elaidic acid (●) on U937 cells. Cells in 96-well plates (2 × 10^4^ cells/well) were incubated with varying concentrations of HOEA or elaidic acid in the growth medium at 37 ℃ for 24 h. The plates were centrifuged, and the supernatants were collected from the wells and conducted LDH assay as described in the text. The results were expressed as % of the total cellular LDH contents. The points indicate the means of triplicate measurements and the bars indicate standard deviation. * indicate significant difference between HOEA and elaidic acid (*p* < 0.05).

To investigate the possibility whether or not HOEA and elaidic acid can affect the plasma membrane integrity and subsequently cause cytolysis, LDH release assay was performed. As shown in Figure 3, HOEA and elaidic acid induced cytolysis in a concentration-dependent manner. The activity of HOEA was much stronger than elaidic acid consistent with CCK-8 assay (Figure 2).

### 2.4. Detection of DNA Fragmentation in HOEA- and Elaidic Acid- Treated U937 Cells

**Figure 4:**
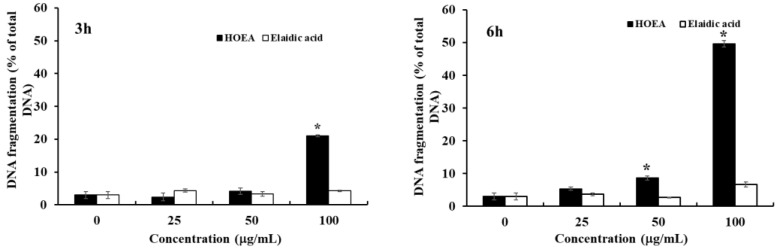
DNA fragmentations in U937 cells treated with HOEA or elaidic acid. Cells (2 × 10^6^ cells/mL) in 35 mm dishes were incubated with indicated concentrations of HOEA (■) or elaidic acid (□) in the growth medium at 37 ℃ for 3 h (left) or 6 h (right), then the extents of DNA fragmentations in the treated cells were examined by diphenylamine assay as described in the text. The data indicate the means of triplicate measurements and the bars indicate standard deviation. * indicate significant difference between HOEA and elaidic acid (*p* < 0.05).

One of the most significant hallmarks of apoptosis is nuclear changes associated with DNA degradation. After the treatment with HOEA or elaidic acid, the levels of fragmented DNA released into cytosol from nucleus were determined by diphenylamine assay. As shown in Figure 4, HOEA induced significant levels of DNA fragmentation in concentration- and time-dependent manners. The activity of elaidic acid was obviously inferior as compared to HOEA. Only slight DNA fragmentation was detected at 100 μg/mL after 6 h incubation.

### 2.5. Nuclear Morophological Changes of U937 Cells Treated with HOEA or Elaidic Acid

**Figure 5:**
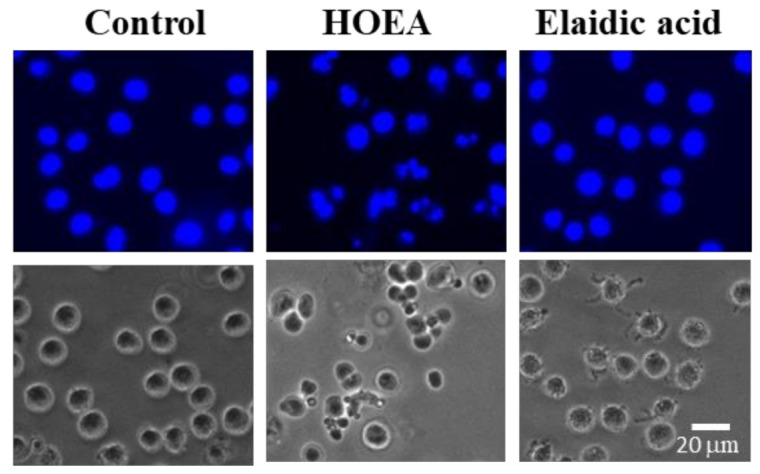
Nuclear morphological changes in U937 cells treated with HOEA or elaidic acid. U937 cells (2 × 10^6^ cells/mL) were incubated without or with 100 μg/mL of HOEA or elaidic acid in the growth medium at 37 °C for 6 h. After Hoechst 33342 staining, the cells were immediately observed by microscope (400× magnification) under fluorescent (upper photographs) and path-contrast condition (lower photographs). Bar indicates 20 μm.

Apoptotic cells generally show characteristic morphological changes such as cell shrinkage, chromatin condensation, and nuclear fragmentation. To analyze apoptotic nuclear morphological changes, HOEA- and elaidic acid- treated cells were stained with Hoechst 33342. As shown in Figure 5, some cells treated with HOEA showed typical apoptotic nuclear morphological changes which were clearly distinctive from the normal cells. The results indicate that the HOEA is capable of inducing apoptotic cell death on U937 cells. Compare to HOEA, no such morphologically changed cells were detected in the cell suspension treated with elaidic acid at least under the conditions used.

### 2.6. Flow Cytometry Analyses of HOEA- and Elaidic Acid-Treated U937 Cells

**Figure 6:**
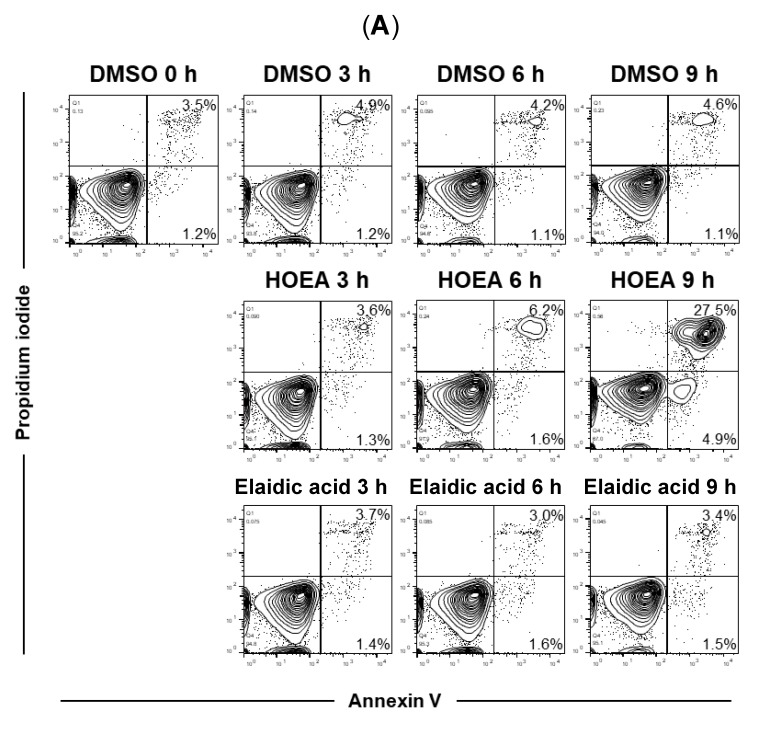

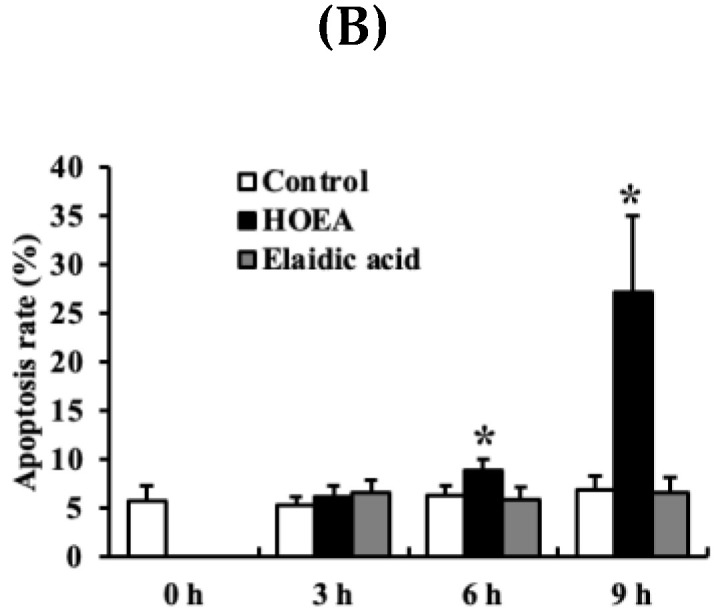
(**A**) U937 cells (5 × 10^5^ cells/mL) were incubated without or with 50 μg/mL of HOEA or elaidic acid in the growth medium at 37 ℃. After 3 h, 6 h and 9 h incubation, the cells were stained with annexin V/PI and analyzed by flow cytometry as described in the text. DMSO alone was used as vehicle for HOEA and elaidic acid. (**B**) The summarized results of time-course analysis of percentage of annexin V+/PI− and annexinV+/PI+ cell populations in the treated cells. The data indicate the means of triplicate experiments and the bars indicate standard deviation. * indicate significant difference between HOEA and elaidic acid (*p* < 0.05).

To further analyze the apoptosis-inducing effect of HOEA, a flow cytometry analysis using the annexin V/PI staining was performed on U937 cells treated with HOEA or elaidic acid. The percentage of apoptotic cells (annexin V+/PI− and annexin V+/PI+) increased from 3.5% to 27.5% in HOEA-treated U937 cells after 9 h incubation, while apoptotic cells detected in control and elaidic acid treated cells was only 4.6% and 3.4%, respectively (Figure 6).

### 2.7. Effects of Caspase-Family Inhibitor (Z-Asp-CH_2_-DCB), N-acetyl-l-cysteine (NAC), and 3-aminobenzamide (3-ABA) on DNA-Fragmentation in HOEA-Treated U937 Cells

**Figure 7:**
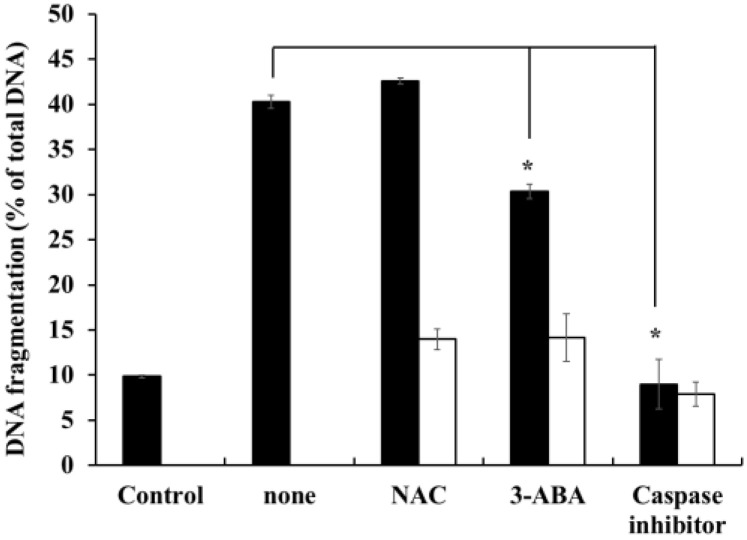
Effects of caspase family inhibitor (Z-Asp-CH_2_-DCB), antioxidant *N*-acetyl cysteine (NAC), and poly (ADP-ribose) polymerase (PARP) inhibitor (3-ABA) on HOEA induced DNA fragmentation in U937 cells. Cells (2 × 10^6^ cells/mL) in 35 mm dish were pre-incubated with Z-Asp-CH_2_-DCB (100 μM), NAC (20 mM) or 3-ABA (20 mM) at 37 ℃ for 1 h, and then HOEA (■) (final 50 μg/mL) was added to the cells. After 3 h of incubation at 37 ℃, the extents of DNA fragmentation of the treated cells were detected by diphenylamine assay as described as the text. The extents of DNA fragmentation in the presence of tested reagent alone without HOEA were measured at the same time (□). The data indicate the means of triplicate measurements and the bars indicate standard deviation. * indicate significant difference between with and without tested reagent (*p* < 0.05).

To gain the tips for HOEA-induced apoptosis mechanism, we examined the effects of Z-Asp-CH_2_-DCB, 3-ABA, and NAC, which are known to affect the apoptosis through acting on the different apoptotic pathways, on HOEA-induced DNA fragmentation in U937 cells. As shown in Figure 7, Z-Asp-CH_2_-DCB, a broad caspase family inhibitor, inhibited the HOEA-induced DNA fragmentation to the almost background level (control). 3-ABA showed slight inhibitory effects but NAC had no effect. These results suggest that caspases are deeply involved in the apoptosis at least at the processes leading to DNA fragmentation. PARP is also suggested to be a regulating factor, but the contribution may be partial.

### 2.8. Effects of Caspase-Family Inhibitor (Z-Asp-CH_2_-DCB) on Annexin V+/PI− and Annexin V+/PI+ Cell Populations in the HOEA-Treated U937 Cells

**Figure 8:**
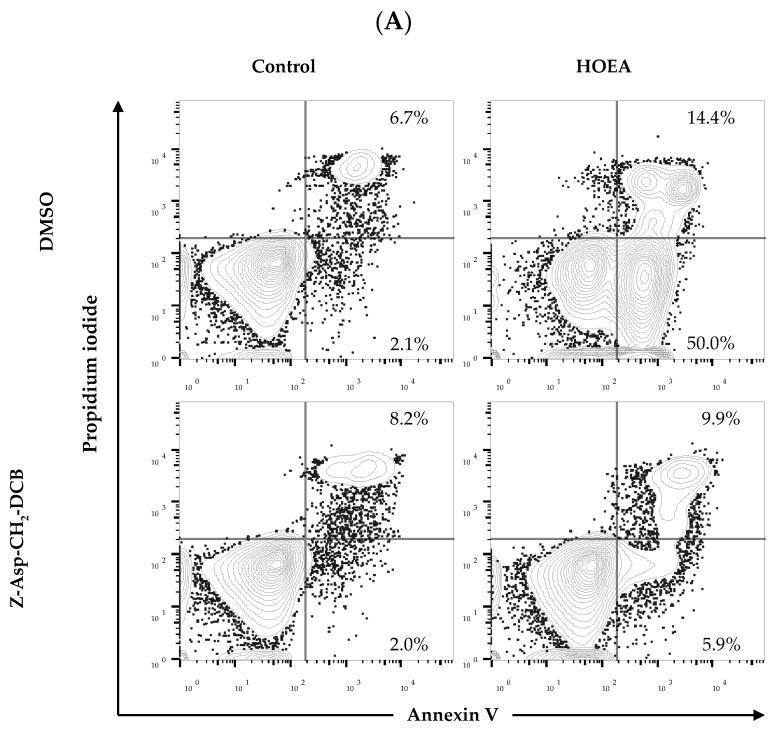

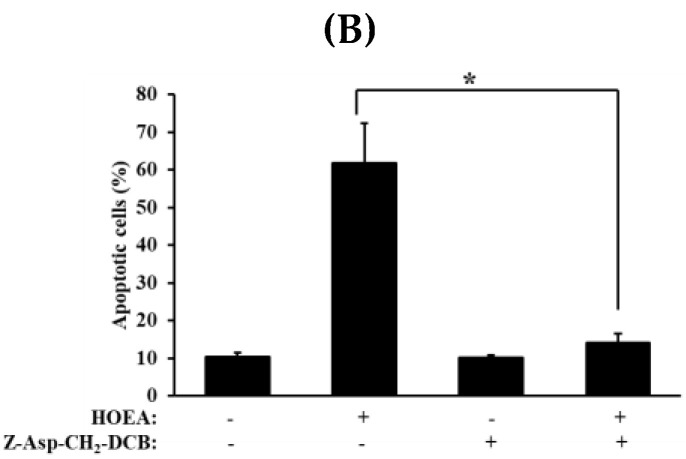
Effects of caspase family inhibitor (Z-Asp-CH_2_-DCB) on HOEA-induced apoptosis detected by flow cytometry. (**A**) U937 cells (5 × 10^5^ cells/mL) were incubated without or with 100 μg/mL of HOEA in the presence or absence of Z-Asp-CH_2_-DCB in the growth medium at 37 ℃. After 6 h incubation, the cells were stained with annexin V/PI and analyzed by flow cytometry as described in the text. (**B**) The summarized results of the annexin V+/PI− and annexin V+/PI+ cell populations. *: *p* < 0.05. The data represent an average of triplicate experiments, and the bars indicate standard deviation.

To further confirm the inhibitory effect of Z-Asp-CH_2_-DCB on the HOEA induced apoptosis in U937 cells, flow cytometry analysis was conducted in U937 cells treated with HOEA in the presence or absence of Z-Asp-CH_2_-DCB. The results clearly indicated that increase in the annexin V+/PI− and V+/PI+ cell populations in HOEA-treated cells was strongly suppressed to almost untreated control level by Z-Asp-CH_2_-DCB (Figure 8).

### 2.9. Analyses of Caspase Activities in HOEA-Treated U937 Cells

**Figure 9:**
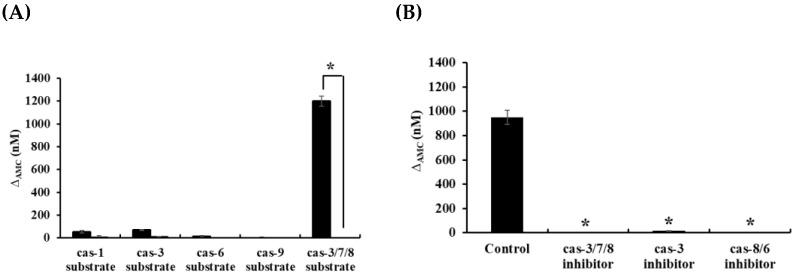
Caspase-like activities in the cytosol of U937 cells treated with HOEA. (**A**) Cells (2 × 10^6^ cells/mL) in 35 mm dish were treated with 100 μg/mL of HOEA (■) or without HOEA (□) in the growth medium at 37 °C for 6 h. Then, the lysates of the treated cells were prepared, and the activities of caspase-1, caspase-3, caspase-9, and caspase-3/7/8 were measured using specific tetra-peptide substrates (10 μM) as described in the text. (**B**) The effects of specific caspase inhibitors (caspase 3, caspase-3/7/8, and caspase-8/6 inhibitors) on the cleavage activities against caspase-3/7/8 substrates in the lysate of HOEA-treated U937 cells were measured as described in the text. The data indicate the means of triplicate measurements and the bars indicate standard deviation. ∆_AMC_ values were calculated by subtraction of 0-time values from the values after 20 min incubation. * in (**A**) indicates significant difference between with and without HOEA (*p* < 0.05). * in (**B**) indicate significant difference between with and without inhibitor (*p* < 0.05).

The aspartate-specific cysteine proteases (caspases) play essential roles in progression of apoptotic cell death at multiple stages [21,22]. Since the potent inhibitory effect of caspase family inhibitor (Z-Asp-CH_2_-DCB) on HOEA-induced DNA fragmentation was observed (Figure 7), it was suggested that certain caspases are involved in the HOEA-induced apoptosis. To examine the activation of caspases in HOEA-induced apoptosis, cytosolic extracts from U937 cells treated with HOEA were incubated with specific fluorescent tetra-peptide substrates for caspase-1, caspase-3, caspase-6, caspase-9, and caspase-3/7/8, respectively. As shown in Figure 9 (A), the cleavage activity against caspase-3/7/8 substrate was drastically increased among the caspases tested. Sight increase in caspase-1 and caspase-3 were detected, but the activities of caspase-6 and caspase-9 were only trace levels. Analyses using three specific inhibitors showed that the increased activity against caspase-3/7/8 substrate in the cell lysate prepared from HOEA-treated U937 cells was markedly inhibited by caspase-3 and caspase-8/6 inhibitors as almost similar extent of the caspase-3/7/8 inhibitor.

### 2.10. Effects of HOEA and Elaidic Acid on the Levels of Cleaved Forms of Caspase 3, Caspase 8, and PARP in U937 Cells

**Figure 10:**
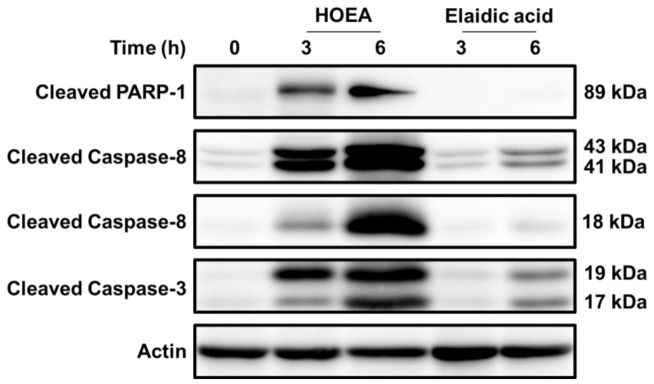
Detection of cleaved forms of caspase 3, caspase 8, and PARP in HOEA or elaidic acid- treated U937 cells. Cells were treated with HOEA or elaidic acid at final 100 μg/mL in the growth medium at 37 ℃ for 0 (control), 3, and 6 h, respectively. The cells were lysed, and then the levels of cleaved forms of the proteins were analyzed with western blotting using specific antibodies for cleaved form of each protein.

To further confirm the activation of caspase cascade in HOEA-treated U937 cells, the levels of cleaved forms of caspase 3, caspase 8, and PARP were analyzed by western blotting. As shown in Figure 10, significant levels of cleaved forms of caspase 8 (p43 and p18) [23] were detected in HOEA-treated U937 cells, and the levels were evidently increased after 6 h incubation as compared to the levels at 3 h. Precursor caspase 3 with 32 kDa (p32) has two cleavage sites, and several cleaved polypeptides with 17 kDa, 12 kDa, 19 kDa, and 29 kDa derived from pro-caspase 3 are produced depending on cleavage processes [24]. In fact, HOEA also produced the cleaved forms of caspase 3 (p17 and p19) especially after 6 h incubation, but elaidic acid was obviously less effective in this regard. Caspase 3 plays a central role in the execution of the apoptosis, and proteolytically activated caspase 3 cleaves several specific substrates including PARP during apoptosis [25,26]. In HOEA-treated cells, cleaved form of PARP (p89) was detected, but such band was undetectable in the cells treated with elaidic acid even after 6 h incubation.

### 2.11. Effect of HOEA on Mitochondrial Membrane Potential in U937 Cells

**Figure 11:**
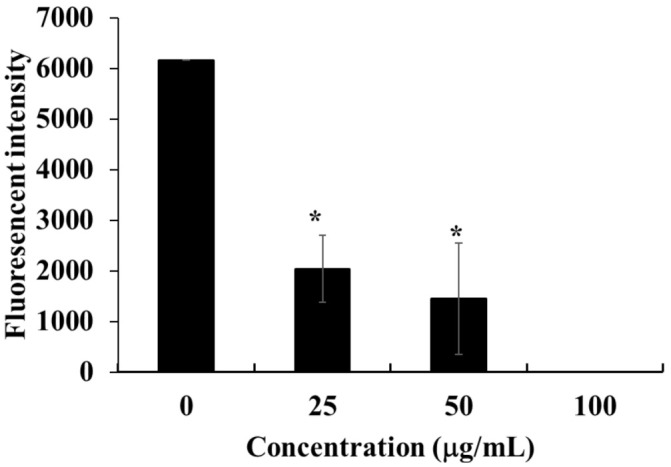
Effect of HOEA on mitochondrial membrane potential in U937 cells. Cells in 96-well plates (4 × 10^4^ cells/well) were incubated with varying concentrations of HOEA at 37 ℃ for 3 h, and then fluorescence probe (DioC6) for mitochondrial membrane potential was added to the treated cells. After 10 min of incubation at 37 ℃, the fluorescence intensities of the cells were measured as described in the text. The data indicate the means of triplicate measurements and the bars indicate standard deviation. * indicate the significant differences between control and sample treated groups (*p* < 0.05).

In the intrinsic apoptosis pathway, also kwon as mitochondrial pathway, certain stimuli change the mitochondrial membrane potential and subsequent increase in the permeability which lead to release of some pro-apoptotic proteins such as cytochrome c [27]. To investigate the possible involvement of the intrinsic pathway in HOEA-induced apoptosis, the mitochondrial membrane potential was measured in HOEA-treated U937 cells using specific fluorescence probe. As shown in Figure 11, treatment with HOEA resulted in marked decrease in the fluorescent intensity in a concentration dependent manner, suggesting that HOEA coursed decrease in the mitochondrial membrane potential.

### 2.12. Cytotoxic Effects of HOEA onVero, CHO, and MDCK Cells

**Figure 12:**
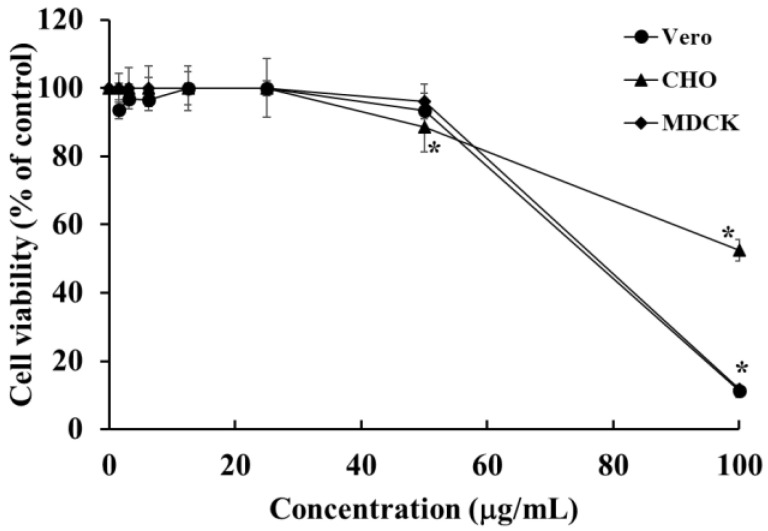
Cytotoxic effects of HOEA on Vero, CHO, and MDCK cells. Adherent cells in 96-well plates (5 × 10^4^ cells/well) were incubated with varying concentrations of HOEA in the growth medium at 37 ℃ for 24 h, and then CCK-8 assay was conducted. The points are means of triplicate measurements and the bars indicate standard deviation. * indicate the significant differences between with and without HOEA (*p* < 0.05).

To clarify the specificity of HOEA action, the cytotoxic effects on Vero, CHO, and MDCK cells as normal tissues-derived cell lines were examined. As shown in Figure 12, CHO cells were highly resistant to HOEA toxicity and the viability was more than 50% even at 100 μg/mL. MDCK and Vero cells also showed relatively lower sensitivity towards HOEA than that of U937 cells. Based on the results obtained in this study and previous our studies [19], estimated LD_50_ values of HOEA against Vero, CHO, MDCK, HeLa, and XC cells were 71.41, >100, 72.81, 75.86, and 84.07 μg/mL, respectively. At least among these cell lines, it seems to be difficult to draw a clear line between normal and tumor cell lines in terms of the susceptibility to HOEA.

### 2.13. Cytotoxic Effect of HOEA on Human Peripheral Blood Mononuclear Cells (PBMCs)

**Figure 13:**
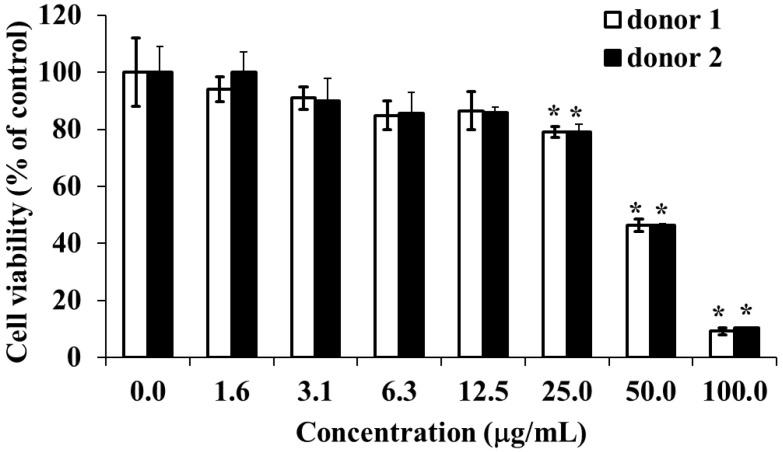
Cytotoxic effect of HOEA on human peripheral blood mononuclear cells (PBMCs). Cells in culture medium in 96-well plates (1 × 10^5^ cells/well) were incubated with varying concentrations of HOEA at 37 ℃ for 24 h, and then CCK-8 assay was conducted. The data show the means of triplicate measurements and the bars are standard deviation. * indicate the significant differences between with and without HOEA (*p* < 0.05).

To examine the effect of HOEA on normal human cells, we used PBMCs perpared from two healthy volunteers. Cells were incubated with varying concentrations of HOEA under normal culture conditions and the viabilities of the cells were asseced by CCK-8 assay. As shown in Figure 13, PBMCs prepared from two doners showed almost equal susceptibility against HOEA in a concentration-dependent manner. Comapred with U937 cells, PBMCs tended to be slightly resistant to HOEA. At the concentration of 50 μg/mL, the viabilitity of PBMCs was nearly 40%, while that of U937 cells was less than 10%.

## 3. Discussion

To seek effective algicidal agents against harmful raphidophycean flagellate *Chattonella antiqua*, we previously conducted extensive screening toward marine macroalgae including more than 15 species of macroalgae such as Rhodophyta, Phaeophyta, and Chlorophyta harvested from the coastal region of Nagasaki Prefecture, Japan. We finally found that the red alga *Tricleocarpa jejuensis* had potent toxic compounds on *C. antiqua* [18]. Chemical structural analyses using NMR, IR, and mass spectral revealed that the active compounds are mixture of four isomers of C18 hydroxy unsaturated fatty acids; (±)-(*E*)-9-hydroxyoctadec-10-enoic acid, (±)-(*E*)-10-hydroxyoctadec-8-enoic acid, (±)-(*E*)-11-hydroxyoctadec-12-enoic acid, and (±)-(*E*)-12-hydroxyoctadec-10-enoic acid. The unambiguously synthesized authentic compounds confirmed the structures. [18]. Several intermediate derivatives were obtained during the processes of the chemical syntheses. Structure-activity relationship analyses on the compounds including synthesized ones suggested that a double bond and a hydroxy group are important structural elements for the algicidal activity [18]. From the four isoforms of C18 hydroxy unsaturated fatty acids which showed nearly similar algicidal activities on *C. antiqua*, (±)-(*E*)-12-hydroxyoctadec-10-enoic acid (HOEA) was selected and further examined its cytotoxic activity against various species. Interestingly, HOEA was found to be cytotoxic to human tumor cells such as XC, HeLa, and U937 cells [19]. Since α-linolenic acid and linoleic acid isolated from green alga *Ulva lactuca* as potent algicidal agents showed no significant cytotoxic effects on mammalian cell lines including HeLa cells and U937 cells [28], the hydroxy group of HOEA may be essential for the antitumor activity. Similar to HOEA, naturally occurring fatty acids show cytotoxic effects on cancer cells [29], and some of them have been recognized as effective antitumor agents [30,31]. Therefore, it seems likely that marine algae are promising resources for finding new fatty acid-related antitumor agents.

In cancer chemotherapy, apoptosis induction in tumor cells is a key to successful therapeutic strategy. Therefore, in this study, we evaluated the cytotoxic mechanism of HOEA on U937 cells especially focusing on the apoptosis inducing ability. To reconfirm the importance of hydroxy group of HOEA, elaidic acid, which is quite similar to HOEA but lacking hydroxy group, was used for comparison. As expected, HOEA showed proliferation inhibitory and cytolytic activities on U937 cells in a concentration-dependent manner, and the activities were much stronger than those of elaidic acid. Nuclear morphological changes associated with DNA degradation is the most significant hallmark of apoptosis. In fact, the cells with typical apoptotic nuclear changes were observed in HOEA-treated cells, but not in elaidic acid-treated cells. Quantitative assay for DNA degradation by diphenylamine assay demonstrated that HOEA induced DNA fragmentation in U937 cells in time- and concentration-dependent manners, while no significant effects of elaidic acid on DNA was observed. Flow cytometric analysis in which cells treated with HOEA were stained with annexin V to detect phosphatidylserine (PS) externalization as another marker of apoptotic cells, indicated that annexin V+/PI− and annexin V+/PI+ cell populations increased in HOEA treated cells, whereas elaidic acid was incapable with respect to such activity. These results clearly indicate that HOEA is capable of inducing potent apoptotic cell death on U937 cells. To our best knowledge, this is the first report indicating that monounsaturated hydroxy C18 fatty acid discovered from *T. jejuensis* can exert apoptosis on U937 cells and importance of hydroxyl group in the activity. Exact way of hydroxy group to involve in the apoptotic cell death activity of HOEA is still unclear, but hydroxy group is generally considered to be highly reactive group interacting with adjustment molecules. Considering the hydrophobic nature of HOEA, the primary cellular target may be plasma membrane. Probably, HOEA can trigger some apoptotic signaling pathways during the processes of hydrophobic interaction with cell membrane and subsequent internalization. The cytolytic effect of HOEA is thought as a result of cell membrane damage. Regarding the cytotoxic mechanism of fatty acid related compounds, it has been reported that a mixture of two unsaturated fatty acids isolated from sponge caused cytotoxicity on mouse Ehrlich carcinoma cells and disruption of erythrocyte membrane at nearly equal 50% effective dose (ED_50_) [32]. The perturbation of the membrane integrity may be a common impact caused by hydrophobic fatty acid-related compounds.

It has been well known that there are two major apoptosis pathways; intrinsic and extrinsic pathways [33]. Caspases (cysteinyl aspartate-specific proteases) play critical roles in controlling apoptosis [21], and activation of multiple different caspases are deeply responsible for the progress of apoptosis depending on the pathways and apoptosis inducers. A broad inhibitor of caspase family proteases, Z-Asp-CH_2_-DCB, is known to block many different apoptotic cell deaths induced by various stimuli including cytotoxic agents [34,35]. In addition to caspases, oxidative stress is also a major mediator of apoptosis, and some antioxidants such as *N*-acetyl cysteine (NAC) blocks apoptosis [36]. Poly(ADP-ribose) polymerase (PARP), a DNA-binding protein activated by DNA strand breaks to participate in DNA repair, is a substrate for caspase-3 or caspase-7 [37]. Exact role of PARP in apoptosis progression is still controversial, but it has been reported that 3-aminobenzamide (3-ABA), a PARP inhibitor, shows an inhibitory effect on apoptosis in several apoptotic cell death models [38,39]. To gain clues to elucidate the apoptosis inducing mechanism of HOEA in U937 cells, the effects of Z-Asp-CH_2_-DCB, NAC, and 3-ABA on HOEA-induced DNA fragmentation in U937 cells were examined. Among these reagents, Z-Asp-CH_2_-DCB showed the most effective inhibitory effect on the DNA fragmentation which was almost control level without HOEA. Only partial inhibitory effect of 3-ABA was observed but NAC was almost no effect. These results prompted us to analyze the caspases activities in HOEA-treated U937 cells. By the analyses using specific tetra-peptide caspase substrates, extremely high cleavage activity against caspase-3/7/8 substrate was detected in HOEA-treated U937 cells, and slight increase in caspase-1 and caspase-3 activity was also found, but caspase-6, and -9 activities were not significant. Since the increased cleavage activity on caspase-3/7/8 substrate was effectively inhibited by caspase-3 and caspase-8/6 inhibitors with equal extend to that of caspase-3/7/8 inhibitor, it was suggested that caspase-3 and caspase-8 mainly contributed to the cleavage activity against caspase-3/7/8 substrate. Activation of caspase cascade involving caspase 3 and 8 in the cells treated with HOEA was also confirmed by western blot analyses. HOEA produced significant levels of cleaved forms of caspase 3 and 8. Cleaved PARP, a primely target of activated caspase 3, was also detected in the cells treated with HOEA. Elaidic acid was obviously less effective in this regard. It has been reported that caspase-8 plays a vital role in executing extrinsic apoptosis induced by ligation of TNF receptor superfamily death receptors [40]. Similar to HOEA, in apoptotic processes induced by quinazoline derivative isolated from marine sponge *Hyrtios erectus*, significant increase in caspase-8 activity was observed in MCF-7 cells treated with the quinazoline derivative [41]. In many apoptosis, caspase-3 plays a pivotal role in downstream executive apoptosis pathways, but MCF-7 cells are lack of caspase-3 expression due to some base-pair deletion of caspase-3 gene [42]. In addition, it has been reported that caspase-7, which has similar substrate specificity to caspase-3, proceeded apoptosis in MCF-7 cells without involvement of casapase-3 [42,43]. These findings suggest that certain caspases other than caspase-3 such as caspase-7 or caspase-8 can take over the role of caspase-3. Since caspase-8 is known as essential initiator caspase of extrinsic death receptor mediated apoptosis [44,45], in the HOEA-induced apoptosis, caspase-8 may involve in the progress of extrinsic apoptosis pathway triggered by the hydrophobic influence of HOEA on plasma membrane. Probably caspase-8 activated by HOEA converges execution phase in which caspase-3 might play a role as an executor, and its direct downstream target is DNase responsible for DNA fragmentation [46]. Further studies are necessary to clarify the primary target molecules of HOEA on the membrane and the way of HOEA to act on it.

To evaluate the possible involvement of the intrinsic pathway also kwon as the mitochondrial pathway in the action of HOEA, change in the mitochondrial membrane potential in U937 cells treated with HOEA was measured. Analysis using fluorescence probe suggested that HOEA remarkably decreased the mitochondrial membrane potential in a concentration dependent manner, suggesting that the mitochondrial pathway was also activated in U937 cells treated with HOEA. Additional studies may provide a clue for the exact mechanism how the mitochondrial pathway is activated by HOEA.

Although the cytotoxic activity of HOEA was different depending on the cell lines used, no clear tendency of tumor cell-specific toxicity of HOEA was observed. Probably, HOEA may not be able to distinguish normal and tumor cells in vitro system using established cell lines and might act on target cells through the commonly existing molecules among these cell lines. To clarify the cytotoxic effect of HOEA on primally cultured normal human cells, PBMCs were used. Although HOEA tended to be slightly less toxic to PBMCs as compared to U937 cells, the significant toxic effect was observed at a high concentration (100 μg/mL). Considering the hydrophobic nature of HOEA as a monounsaturated fatty acid, it is reasonable to speculate that cell membrane might be a primary target of HOEA, and it seems unlikely to exert cytotoxicity in a tumor specific manner at least when it acts on target cells directly in in vitro system. These results suggest that HOEA can cause adverse effects on normal cells while killing cancer cells in vivo at the effective doses. Cancer chemotherapy usually relies on the premise that actively proliferating tumor cells are more likely to be affected by cytotoxic agents as compared to normal cells. Unfortunately, HOEA does not seem to fulfill this criterion. However, not limited to HOEA, the severe side effects of the cytotoxic drugs on normal cells and tissues are major problems of cancer chemotherapy [47,48]. Therefore, developing various drug delivery procedures and systems is a promising approach to overcome the drawbacks and improve the therapeutic efficacy of chemotherapy. When it comes to effective drug delivery systems, the molecular size of drugs should be taken into consideration as an important factor influencing the pharmacokinetics. Regarding physiological characteristics of tumor tissues, it has been known that blood vessels in solid tumor tissues show characteristic features different from those of normal tissues [49], and demonstrated that multiple tumor-specific factors enhance the tumor-selective vascular permeability of large molecular weight substances [49,50], and such large molecules tend to remain in tumor tissues for long periods due to the lack of sufficient lymphatic recovery system in tumors [49,50]. The phenomenon of enhanced permeability and retention is known as the EPR effect [49,50,51]. Many of the conventional cancer chemotherapeutic agents are low molecular chemicals and are distributed throughout the body [52] when they are injected intravenously. That can be the primary reason for adverse side effects on normal tissues. In contrast, high molecular weight molecules tend to selectively accumulate in tumor tissue due to the EPR effect. At the present, the pharmacokinetics of HOEA in human body is unclear, but it may hardly exist as free form in the circulation. Probably HOEA binds to certain serum components such as albumin through the hydrophobic interaction as seen in other fatty acids [53]. Such complexed HOEA may behave like macromolecules, which can utilize the EPR effect. Further studies are obviously necessary for in vivo analyses on the pharmacokinetics of HOEA.

Regarding bioactive unsaturated fatty acids (USFs) like HOEA, previous studies have found that USFs have versatile great potential in cancer chemotherapy due to their certain antitumor activities, the ability to increase the chemotherapeutic sensitivity of other antitumor drugs, the good biocompatibility, and even natural tumor-targeting properties [54,55,56]. Taking the advantages of fatty acids, several conjugates between fatty acids and drugs have been constructed by chemical modification procedures to improve the chemotherapeutic efficacy [57]. Furthermore, combination between chemotherapy agent-unsaturated fatty acids conjugated prodrugs and nanoparticle-drug delivery technology have been reported as clinically applicable cancer therapy [54,58]. HOEA may be utilized as a counterpart of the conjugation with therapeutic drugs or component of nanoparticle-based chemotherapy.

In conclusion, we found that HOEA, a C18 monounsaturated hydroxy fatty acid isolated from red alga *T. jejuensis*, was capable of inducing apoptotic cell death in U937 cells through the activation of extrinsic and intrinsic pathways in which especially caspase-3 and caspase-8 may play pivotal roles, and hydoxy group was a key structural element for the activity. Since the cytotoxic action of HOEA is not strictly tumor cell-specific, it is essential to develop the tumor-selective delivery systems for the clinical applications.

## 4. Materials and Methods

### 4.1. Chemicals

HOEA and elaidic acid were prepared as reported previously [18]. The stock solutions of HOEA and elaidic acid in dimethyl sulfoxide (DMSO) at 4 mg/mL were prepared and stored at 4 °C. The structures are shown in Figure 1. The fluorescent tetrapeptide specific substrate for caspase-1 (Ac-Tyr-Val-Ala-Asp-MCA), caspase-3 (Ac-Asp-Asn-Leu-Asp-MCA), caspase-6 (Ac-Val-Glu-Tle-Asp-MCA), caspase-9 (Ac-Leu-Glu-His-Asp-MCA) and caspase-3/7/8 (Ac-Asp-Glu-Val-Asp-MCA), and specific caspase-3 inhibitor (Cbz-Asp-Glu-Val-Asp-CH_2_F), caspase-3/7/8 inhibitor (Ac-Asp-Glu-Val-Asp-H), caspase-8/6 inhibitor (Ac-Ile-Glu-Thr-Asp-H), and broad spectral caspase family inhibitor (Z-Asp-CH_2_-DCB), and fluorescence standard 7-amino-4-methylcoumarin (AMC) were obtained from Peptide Institute, Osaka, Japan.

### 4.2. Cell Culture

U937 cells obtained from Riken Cell Bank (Tsukuba, Japan) were cultured in RPMI 1640 medium supplemented with 10% fetal bovine serum (FBS), penicillin (100 IU/mL), and streptomycin (100 μg/mL) at 37 °C in a humidified atmosphere with 5% CO_2_ and 95% air. MDCK (Madin-Darby canine kidney), Vero (African green monkey kidney), and CHO (Chinese hamster ovary) cells obtained from the American Type Culture Collection (Rockville, MD, USA) were cultured in α-MEM supplemented with 10% fetal bovine serum (FBS), penicillin (100 IU/mL), and streptomycin (100 μg/mL) at 37 °C in a humidified atmosphere with 5% CO_2_ and 95% air.

### 4.3. Cytotoxicity Assay

Effects of HOEA and elaidic acid on the viability of U937 cells were measured by a Cell Counting Kit-8 (CCK-8) purchased from (Dojindo, Kumamoto, Japan) according to the manufacturer’s instructions. In brief, the cells in 96-well plates (2 × 10^4^ cells/well) were incubated in the growth medium with varying concentrations of HOEA or elaidic acid at 37 °C for 24 h. Then, 10 μL of the CCK-8 solution was added to each well of the plates, and the plates were incubated for 2 h at 37 °C. The absorbance was measured at 450 nm using a microplate reader (MPR-A4i, TOSOH Co., Ltd., Tokyo, Japan). Effects of HOEA on MDCK, Vero, and CHO cells were examined by basically the same procedure.

### 4.4. Cytolytic Assay

The cytolytic activity of HOEA and elaidic acid was estimated by the lactate dehydrogenase (LDH) assay as described previously [59]. In brief, the cells (2 × 10^4^ cells/well) in 96-well plates were incubated in the growth medium with varying concentrations of HOEA or elaidic acid. After incubation at 37 °C for 24 h, the plates were centrifuged at 1500 rpm at 4 °C for 20 min. The supernatant (50 μL) was collected from each well, and then the supernatant was incubated with an equal volume of working solution at room temperature for 30 min. The reaction was stopped by the addition of 25 μL of stop solution and then the absorbance was measured at 490 nm using a microplate reader (MPR-A4i, TOSOH Co., Ltd., Tokyo, Japan). The results were expressed as the percentage release of total cellular LDH levels.

### 4.5. Fluorescence Observation of Nuclear Morphological Changes

Cells (2 × 10^6^ cells/mL) in the growth medium were incubated with HOEA or elaidic acid at the indicated concentrations at 37 °C. After 3 h incubation. the cells were stained with Hoechst 33342 (10 μg/mL) for 15 min under 37 °C and observed by a fluorescence microscope (Keyence BZ-X710, Osaka, Japan).

### 4.6. Quantification of DNA Fragmentation

DNA fragmentation was measured by diphenyl amine assay as described previously [59]. Cells (2 × 10^6^ cells/dish) in the growth medium were incubated with HOEA or elaidic acid at the indicated concentrations at 37 °C for 3 h and 6 h, respectively. The cells were harvested by centrifugation (2000 rpm for 10 min at 4 °C) and lysed in 300 μL of ice-cold lysis buffer (0.5% Triton X-100, 10 mM pH 7.4 Tris-HCl, 20 mM pH 8.0 EDTA· 2Na). The cell lysates were centrifuged for 20 min at 15,000 rpm to separate DNA fragments (supernatant) from intact DNA (pellet). The DNA contents of the supernatant and pellet fractions were determined using diphenylamine reagent. The results were expressed as % fragmentation per total cellular DNA contents.

### 4.7. Measurement of the Effects of Caspase Family Inhibitor (Z-Asp-CH_2_-DCB), NAC and 3-ABA on HOEA-Induced DNA Fragmentation in U937 Cells

Cells (2 × 10^6^ cells/mL) in 35 mm dish in the growth medium were incubated with 100 μM caspase family inhibitor (Z-Asp-CH_2_-DCB), 20 mM *N*-acetyl-l-cysteine (NAC), or 20 mM 3-aminobenzamide (3-ABA) at 37 °C for 1 h, and then the indicated concentrations of HOEA or elaidic acid was added to the cells. After 3 h incubation at 37 °C, the extents of DNA fragmentation of the treated cells were measured by diphenyl amine assay.

### 4.8. Caspase Activities

Cells (2 × 10^6^ cells/dish) in the growth medium were incubated with the indicated concentrations of HOEA at 37 °C for 3 h, then the cells were harvested by centrifugation (2000 rpm, for 8 min) and washed with PBS once, then lysed in 300 μL of extraction buffer (10 mM HEPES/KOH buffer, pH7.4, 2 mM EDTA·2 Na, 0.1% CHAPS, 5 mM DTT, 1 mM PMSF) as described [35]. After repeated freezing and thawing, cell debris was removed by centrifugation at 15,000 rpm at 4 °C for 20 min. The supernatants were incubated with 10 μM of caspase-specific peptide substrates at 37 °C for 20 min, then the increase in the fluorescence intensities reflecting the substrate cleavage activities were measured with excitation at 380 nm and emission at 460 nm. The concentrations of AMC released from the substrates by the cleavage reaction during the incubation periods were calculated based on the known concentration of AMC standard.

### 4.9. Flow Cytometry Analysis

Apoptotic cells were also analyzed by annexin V and propidium iodide (PI) staining. The cells were incubated with the indicated concentrations of HOEA or elaidic acid in the growth medium for the indicated period of time at 37 °C, and then the cells were harvested and washed once with PBS by centrifugation (1500 rpm for 5 min). After staining with Pacific Blue^TM^ annexin V (BioLgened, 640918) and PI for 15 min at room temperature in the dark, the stained cells were analyzed on FACSCelesta (BD Bioscience, San Jose, CA, USA). Data were analyzed on FlowJo software version 10.4 (Tree Star, San Carlos, CA, USA). The entire proportion of apoptotic cells is determined as the total of early and late apoptotic cells, which was detected as the percentage of annexin V+/PI− and annexin V+/PI+ cells, respectively.

### 4.10. Western Blotting

U937 cells in 12-well plates (2 × 10^6^ cells/well) were treated with 100 μg/mL of HOEA or Elaidic acid for the indicated periods of time in the growth medium at 37 °C, and then the cells were lysed in lysis buffer (25 mM HEPES, 10 mM Na_2_P_2_O_7_∙10H_2_O, 1% Triton X-100, 5 mM EDTA∙2Na, 100 mM NaF, 2 mM Na_3_VO_4_) containing 1% protein inhibitor cocktail (Nacalai Tesque Inc., Kyoto, Japan) on ice for 1 h. After removing the insoluble materials by centrifugation (15,000 rpm for 15 min at 4 °C), the cell lysate (20 μg protein/lane) was subjected to sodium dodecyl sulfate-polyacrylamide gel electrophoresis. The proteins were then electrophoretically transferred to an Immobilon-P polyvinylidene difluoride membrane (Merck Millipore, Burlington, MA). Separated proteins on the membranes were analyzed by western blotting using following specific primary antibodies; anti-cleaved caspase-3 (9661), anti-cleaved caspase-8 (9496) (Cell Signaling Technology, Danvers, MA, USA), anti-actin (sc-8432), anti-cleaved PARP-1 (sc-56196, Santa Cruz Biotechnology, Santa Cruz, CA, USA). To detect specifically bound primary antibodies, the blots were incubated with horseradish peroxide-labeled secondary antibodies, and were visualized using Chemi-Lumi One Super (Nacalai Tesque Inc., Kyoto, Japan) and an ImageQuant LAS4000 system (GE Healthcare, Little Chalfont, UK).

### 4.11. Detection of Mitochondrial Membrane Potential

Cells (4 × 10^4^ cells/well) in 96-well plates in the growth medium were incubated with varying concentrations of HOEA at 37 °C for 3 h. The cells were washed with PBS once and then incubated with 40 nM 3,3′-dihexyloxacarbocyanine iodide (DioC6, Sigma, Inc, St. Louis, MO, USA) for 30 min. The fluorescence intensities of the cells were measured at excitation wavelength of 488 nm and emission wavelength of 525 nm.

### 4.12. Preparation of PBMCs and Cell Viability Assay

In accordance with the Declaration of Helsinki, approval for this study was obtained from Kumamoto University medical ethics committee (Approval No. 548, 1 February, 2021). Informed consent was obtained for the collection of peripheral blood from healthy human donors (2 donors). Peripheral blood mononuclear cells (PBMCs) were separated using Pancoll Human (PAN Biotech, Geschäftszeiten, Germany) from blood donated by healthy donors. Isolated PBMCs in 96-well plates (1 × 10^5^ cells/well) were cultured with varying concentrations of HOEA at 37 °C in RPMI-1640 supplemented with 10% FBS, penicillin (100 IU/mL), and streptomycin (100 μg /mL) in a humidified atmosphere containing 5% CO_2_ and 95% air. After 24 h incubation, the cell viabilities were measured by CCK-8 assay as described above.

### 4.13. Statistical Analysis

All the experiments were conducted at least three times. The results were expressed as mean ± standard deviation (SD). The data were analyzed by one-way ANOVA test to determine the significant difference by using Graphpad Prism 6 software (GraphPad, San Diego, CA, USA). The value of *p* < 0.05 was considered statistically significant.

## Data Availability

Data is contained within the article. The data presented in this study are available in Supplementary Mterials.

## Supplementary Materials

The following are available online at https://www.mdpi.com/1660-3397/19/3/138/s1, Figures S1–S3: Flow cytometry data of Figure 6; Figure S4: Flow cytometry data of Figure 8.
