# Supplementary material for: Induction of Apoptotic Cell Death in Human Leukemia U937 Cells by C18 Hydroxy Unsaturated Fatty Acid Isolated from Red Alga Tricleocarpa jejuensis
Source: Mar Drugs. 2021 Mar 2;19(3):138. doi: 10.3390/md19030138 (PMC8001238; doi:10.3390/md19030138)
Supplement: Supplementary file 1 [file marinedrugs-19-00138-s001.pdf]

SUPPORTING INFORMATION

# Induction of Apoptotic Cell Death in Human Leukemia U937 Cells by C18 Hydroxy Unsaturated Fatty Acid Isolated from Red Alga *Tricleocarpa jejuensis*

**Shijiao Zha [1,†], Mikinori Ueno [2,†], Yan Liang [1], Seiji Okada [2], Tatsuya Oda [1] and Fumito Ishibashi [1,*]**

[1]Graduate School of Fisheries and Environmental Sciences, Nagasaki University, 1-14 Bunkyo-machi, Nagasaki 852-8521, Japan; bb53418001@ms.nagasaki-u.ac.jp (S.Z.); bb53419804@ms.nagasaki-u.ac.jp (Y.L.); t-oda@nagasaki-u.ac.jp (T.O.)

[2]Joint Research Center for Human Retrovirus Infection, Division of Hematopoiesis, Kumamoto University, 2-2-1 Honjo, Chuo-ku, Kumamoto 860-0811, Japan; mikiueno@kumamoto-u.ac.jp (M.U.); okadas@kumamoto-u.ac.jp (S.O.)

*****Correspondence: fumito@nagasaki-u.ac.jp; Tel.: +81-95-819-2833

[†] Both authors have contributed equally to this work

## Contents

Flow cytometry data of Figure 6 S1~S3
Flow cytometry data of Figure 8 S4

# Flow cytometry data of Figure 6

First time

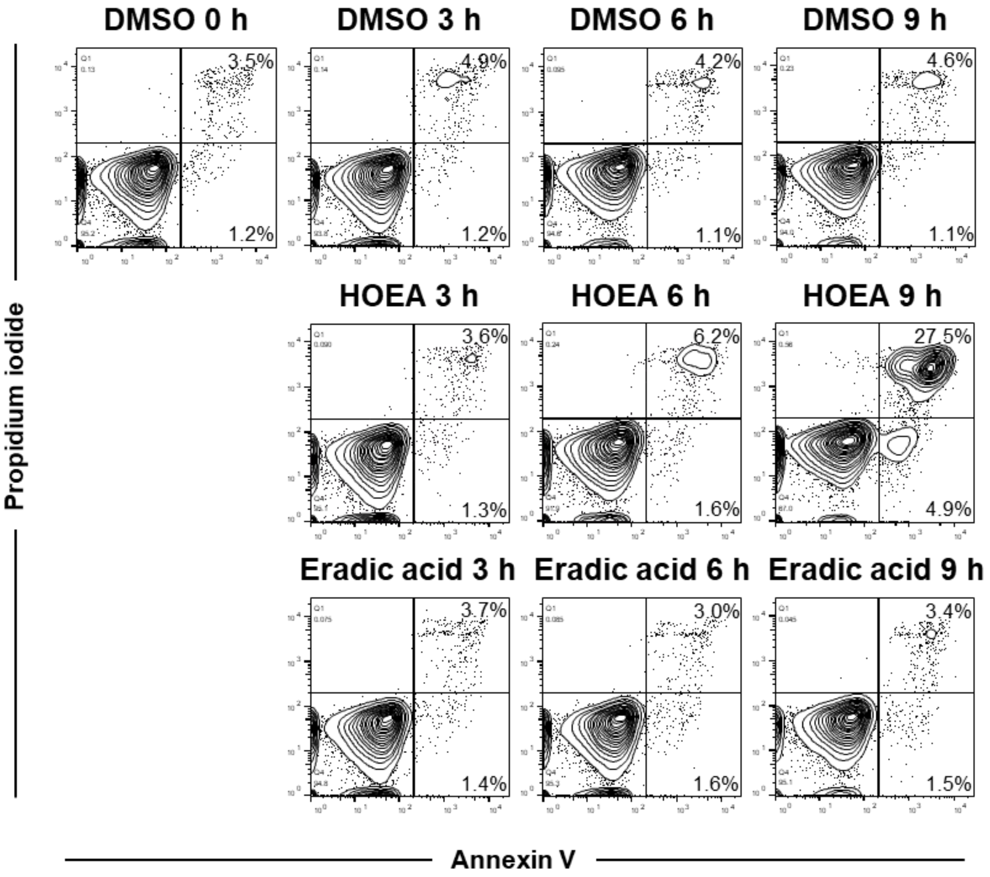

# Flow cytometry data of Figure 6

Second time

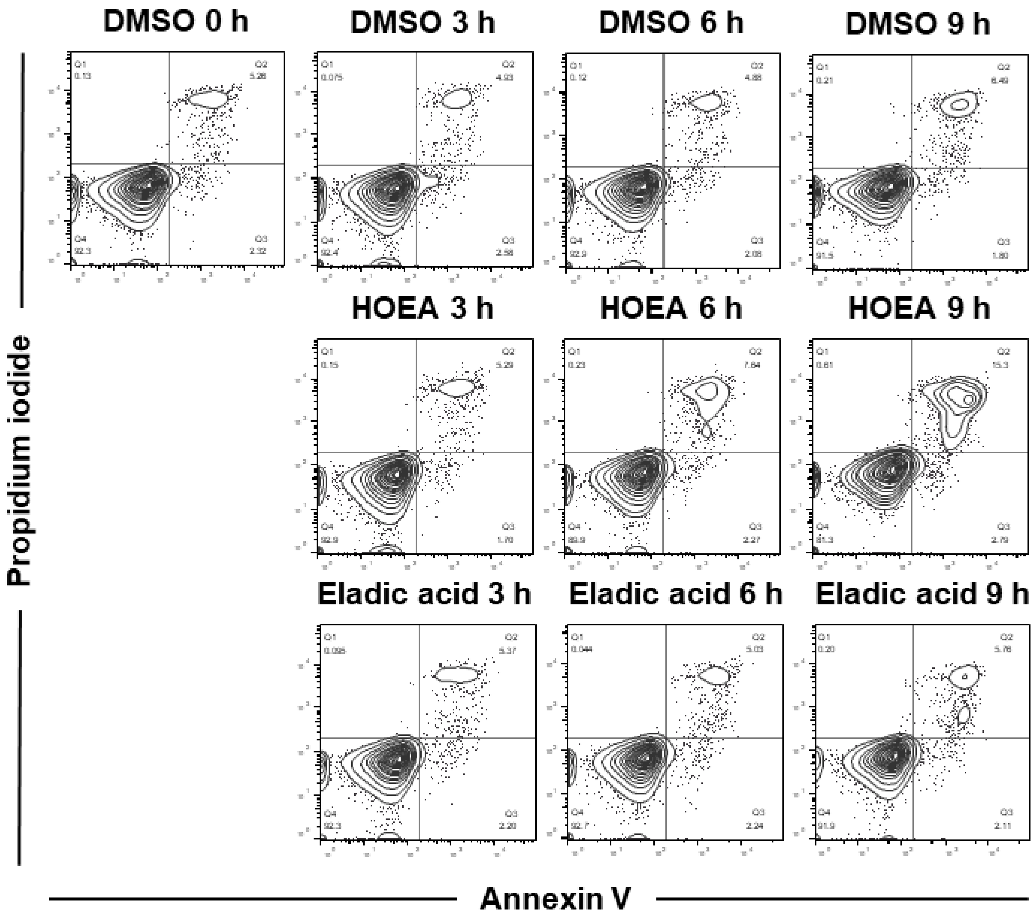

# Flow cytometry data of Figure 6

Third time

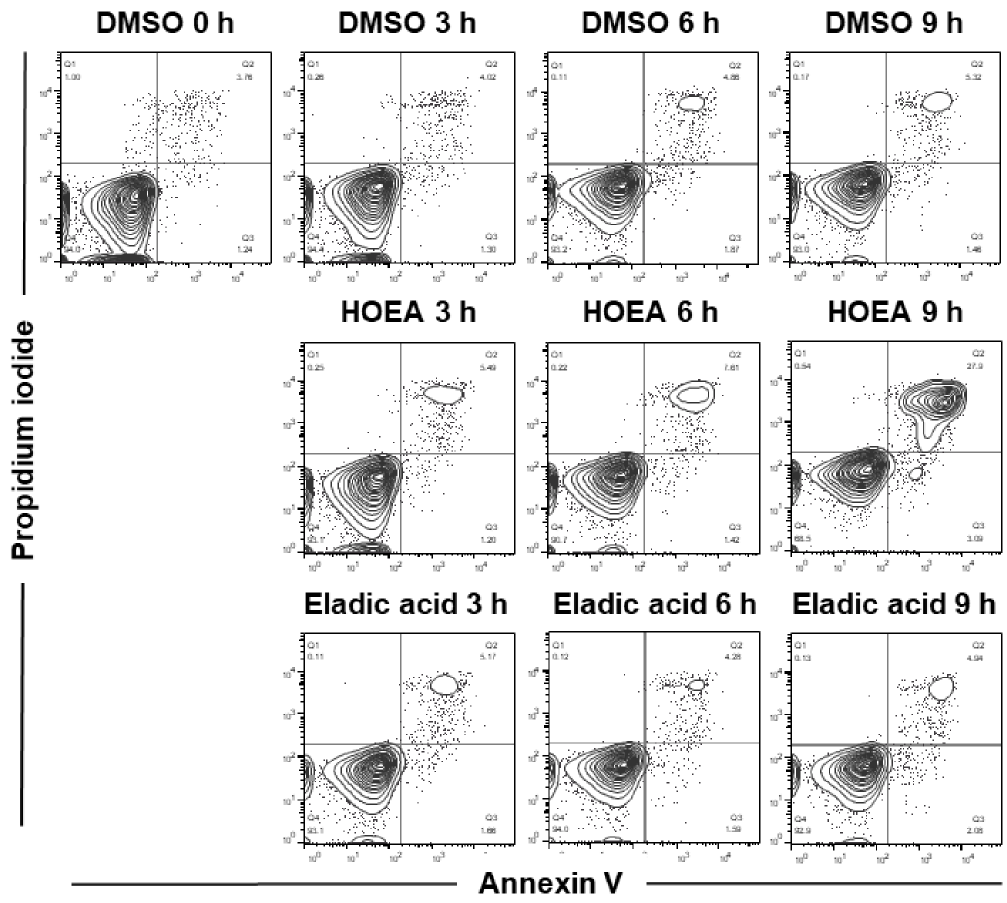

## Flow cytometry data of Figure 8

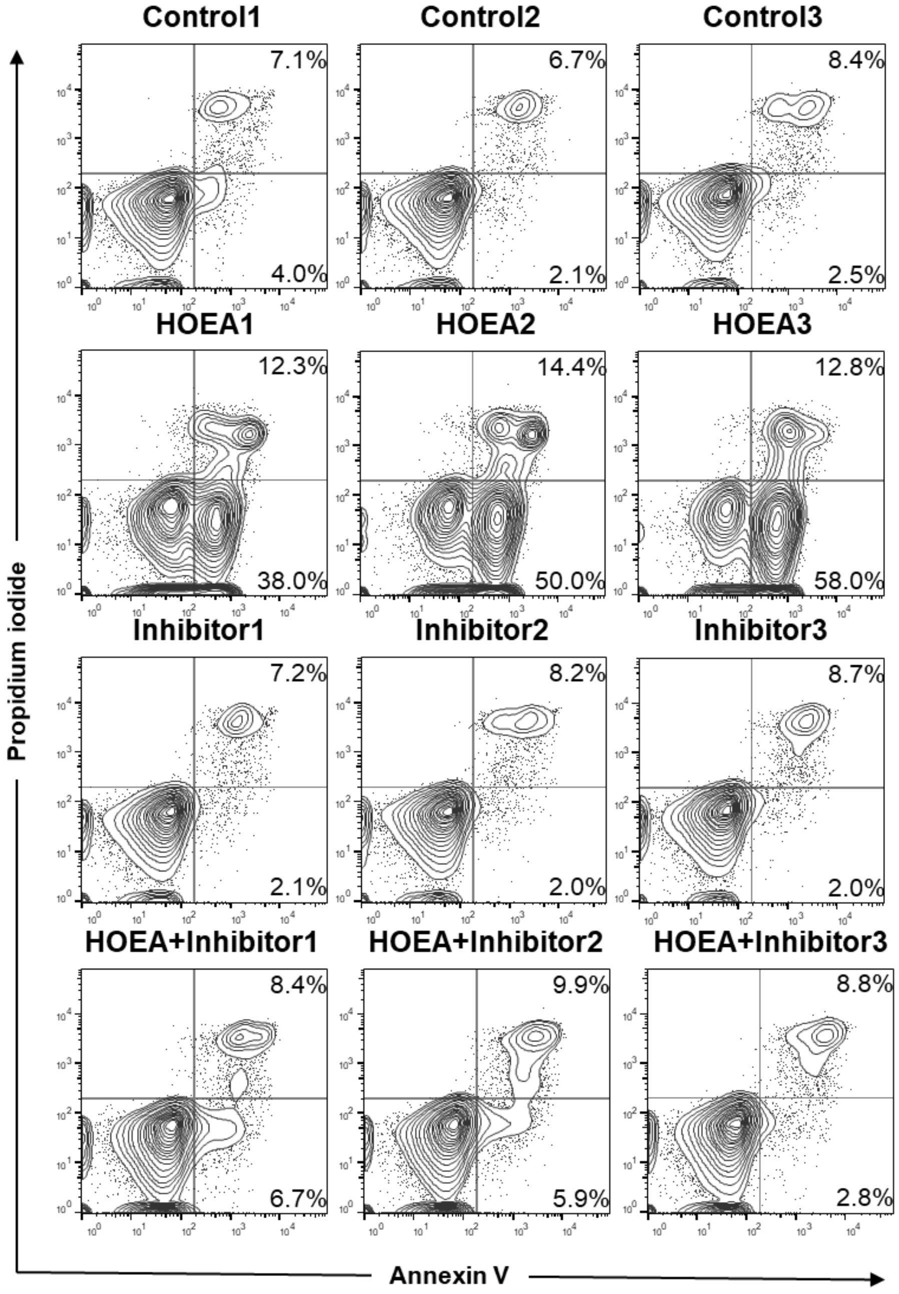


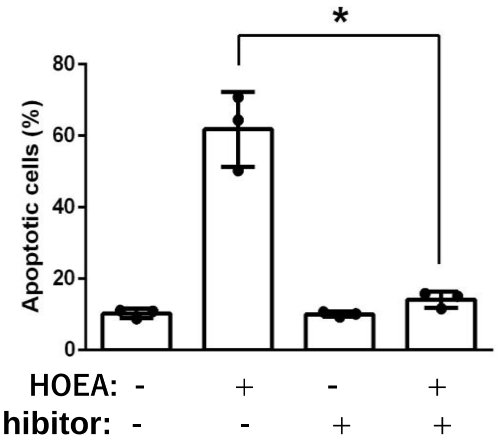


Inhibitor: Z-Asp-$CH_2$-DCB
